# Child-Report of Food Insecurity Is Associated with Diet Quality in Children

**DOI:** 10.3390/nu11071574

**Published:** 2019-07-12

**Authors:** Matthew J. Landry, Alexandra E. van den Berg, Fiona M. Asigbee, Sarvenaz Vandyousefi, Reem Ghaddar, Jaimie N. Davis

**Affiliations:** 1 Department of Nutritional Sciences, The University of Texas at Austin, Austin, TX 78723, USA; 2 Michael & Susan Dell Center for Healthy Living, Division of Health Promotion and Behavioral Sciences, University of Texas Health Science Center at Houston (UTHealth, School of Public Health in Austin, Austin, TX 78701, USA

**Keywords:** food security, food insecurity, diet quality, diet patterns, children, healthy eating index

## Abstract

Food insecurity (FI) is adversely associated with physical and mental wellbeing in children. The mechanism underlying this association is assumed to be dietary intake; however, evidence has been mixed. This study examined the relationship between self-reported FI and dietary quality among low-income children. Cross-sectional data were used from TX Sprouts, a school-based cooking, gardening, and nutrition intervention. A sample of 598 children completed two 24-h dietary recalls and a questionnaire including an adapted version of the 5-item Child Food Security Assessment (CFSA). Food security was categorized as food secure or FI based on summed CFSA scores. Dietary quality was assessed using the Health Eating Index-2015 (HEI-2015). Mixed effects linear regression models examined associations between FI and dietary quality. Children were 64% Hispanic, 55% female, and were 9.2 years old on average. Adjusting for sociodemographic characteristics, BMI percentile, and energy intake, FI was associated with lower HEI-2015 total scores (β = −3.17; 95% CI = −5.28, −1.06; *p* = 0.003). Compared to food secure children, FI children had lower greens and beans (2.3 vs. 1.9, *p* = 0.016), seafood and plant protein (2.0 vs. 1.6, *p* = 0.006), and added sugar (7.4 vs. 8.0, *p* = 0.002) component scores. Interventions targeting low-income and FI children should investigate ways to improve dietary quality.

## 1. Introduction

In 2017, children in 7.7% of United States (U.S.) households (approximately 2.9 million households) lived within food-insecure households, meaning that their household access to adequate food was limited by a lack of money and other resources [1]. The health consequences of child food insecurity are well documented [2]. The mechanism that underlies food insecurity contributing to poor health is assumed to be unhealthy dietary intake. Because of limited time and resources, food insecurity may contribute to, or exacerbate, poor dietary intake; however, evidence linking food insecurity to child dietary intake is unclear [3,4].

A 2014 systematic review by Hanson and Connor examined 16 articles and 130 associations between food insecurity and dietary intake in children [3]. Of the 130 associations, 16% suggested an adverse association, 3% suggested a beneficial relationship, and the remaining indicated a nonsignificant, ambiguous, or inconsequential association. These studies primarily emphasized the relationship of single macronutrient, micronutrients, or individual foods or food groups in diet-food insecurity relationships. More recently, researchers have focused less on the associations of individual nutrients or foods in isolation with disease risk and have examined a more inclusive approach to diet and health using dietary patterns [5,6]. Diet patterns focus on the synergy of nutrients within the context of total dietary intake, and can be used for assessing individual contributions of dietary components on health outcomes simultaneously. Dietary indices such as the Healthy Eating Index (HEI) [7], which measures adherence to the U.S. Dietary Guidelines for Americans (DGA), have been associated with numerous chronic diseases [8].

Of the three studies that included measures of overall diet quality to examine the association with child food insecurity, results were split between adverse associations and no associations [9,10,11]. More recently, three studies reported no association between dietary quality and child food insecurity [12,13,14]. A limitation of nearly all previously mentioned studies examining the relationship between food insecurity and dietary quality is the use of parent-reported household food insecurity [3]. Consistently, research has shown that parental report of child-level food insecurity may be unrepresentative of actual child food insecurity experiences and if a parent proxy is used, prevalence of child-level food insecurity may be grossly underestimated [15,16,17,18,19]. The two studies that have used child self-report of their own food security experiences to examine associations between dietary quality and food insecurity have found no association [12,13]. However, these studies were limited in that they utilized a geographically limited sample and did not control for body weight or body mass index (BMI) as a potential confounding variable [12,13].

The present study sought to use a child’s report of their food insecurity experiences to examine the relationship between food insecurity and dietary quality in a multiethnic cohort of children (7–13 years of age). Food insecurity disproportionally impacts non-Hispanic Blacks and Hispanics compared to non-Hispanic Whites [1]. Whereas previous studies have stratified their sample based on *a priori* hypotheses of demographic differences, this study sought to examine the interaction between sex and ethnicity/race and food insecurity prior to stratification. 

## 2. Materials and Methods

### 2.1. Description of Study

Cross-sectional baseline data from TX Sprouts, a cluster-randomized school-based gardening, cooking, and nutrition intervention, were used. TX Sprouts targeted 3rd–5th grade students and their parents from 16 elementary schools in the Austin area. Schools were randomized into one of three waves of data collection occurring between August 2016 and October 2018. Schools included in the trial had to meet the following inclusion criteria: (1) high proportion of Hispanic children (>50%); (2) high proportion of children participating in the free and reduced lunch (FRL) program (>50%); (3) location within 60 miles of (university name omitted for blind peer review) campus; and (4) no previous or existing gardening program. Based on these criteria, 73 schools were invited to participate, and 20 schools from five different independent school districts agreed to participate. There were no statistical differences in the proportion of Hispanic children and proportion of children participating in FRL in schools that agreed to participate compared to those that did not participate. The first 16 out of the 20 schools to provide letters of support were randomly assigned to either intervention (*n* = 8 schools) or control group (delayed intervention; *n* = 8 schools). Full methods of the ongoing TX Sprouts intervention will be published elsewhere. The trial is registered at ClinicalTrials.gov (trial number omitted for blind peer review).

### 2.2. Study Recruitment

All 3rd–5th grade students and parents at the recruited schools were contacted to participate via tables at “Back to School” and “Meet the Teacher” evenings events, flyers that were sent home with students, and teachers making class announcements. 

### 2.3. Institutional Review Board

Written informed consent was obtained from all parents, and assent from each student was obtained. Both consent and assent were required for inclusion in the study. This study was conducted according to the guidelines laid down in the Declaration of Helsinki and all procedures involving human subjects were approved by the Institutional Review Boards of (University Name Omitted for Blind Peer Review) and the individual school district review boards. 

### 2.4. Data Collection

At baseline, children completed a 12-page questionnaire packet that included items about demographics and a food security scale. Students completed all questionnaires during the school day at their respective schools as part of a larger data collection effort for TX Sprouts. Questionnaires were provided in both English and Spanish, and bilingual interpreters were available to assist students if needed. Parents completed a separate 12-page self-administered questionnaire that was provided in both English and Spanish. Parents received a $15 gift card to a local grocery store as an incentive for completing the questionnaire. 

Anthropometric measurements were collected on children. Height was measured using a free-standing stadiometer to the nearest 0.1 cm (Seca, Birmingham, UK). Waist circumference was measured using National Health and Nutrition Examination Survey (NHANES) protocol [20]. Weight and bioelectrical impedance were assessed with the Tanita Body Fat Analyzer (Tanita Corporation of America Inc, IL, USA, model TBF 300). BMI percentiles were determined using Centers for Disease Control and Prevention (CDC) age- and gender-specific values [21].

### 2.5. Dietary Intake

Sixteen students (eight male and eight female) were randomly selected from each grade level at each school (for a total of 48 students/school) to be contacted for 24-h dietary recalls. If any of the 16 students were not available or did not want to participant in recalls, then additional students were randomly selected to fill in as back-ups. Each student completed two 24-h dietary recalls. Recalls were collected via telephone by trained staff and supervised by a Registered Dietitian Nutritionist using the Nutrition Data System for Research (Nutrition Coordinating Center; 2016) [22], a computer-based software application that facilitates the collection of recalls in a standardized fashion [23]. Dietary intake data gathered by interview was governed by a multiple-pass interview approach [24]. Five distinct passes provided multiple opportunities for the participant to recall food intake. Students took approximately 20 to 30 min to complete each recall. A Food Amounts Booklet was distributed to students and used to estimate serving sizes during recalls. Menus and portion sizes were obtained from school food services to aid in collecting recalls. Parents and/or guardians of students were allowed to assist with recalls as needed. Assistance included recalling food items consumed and estimating serving sizes. Students received a $10 incentive for completing the recalls. Quality assurance was performed on all dietary recall data by additional trained research staff. 

### 2.6. Calculation of the Healthy Eating Index-2015

Diet quality was assessed using the Healthy Eating Index-2015 (HEI-2015). The HEI-2015 is a valid and reliable composite measure that helps assess overall diet quality and compliance with the Dietary Guidelines for Americans-2015 (DGA-2015) [25,26]. The index is appropriate for examining diet quality of the U.S. population as well as specific subgroups such as children and adolescents or racial-ethnic populations in a range of applications including epidemiology, population monitoring and surveillance, and nutrition interventions [7]. The HEI-2015 is based on thirteen components (total fruit, whole fruit, total vegetables, greens and beans, whole grains, dairy, total protein foods, seafood and plant proteins, fatty acids, refined grains, sodium, saturated fats, and added sugars) [27]. The first nine components are adequacy scores, with higher scores indicating higher consumption, and scores of zero indicating no intake. The remaining four components (refined grains, sodium, saturated fat, and added sugars) are components for moderation. For moderation components, reverse scoring is applied, meaning that higher scores indicate lower consumption. Total fruit, whole fruit, total vegetables, greens and beans, total protein foods, seafood and plant proteins have a maximum score of five, and whole grains, dairy, fatty acids, refined grains, sodium, saturated fats, and added sugars which have a maximum score of 10. A total HEI score can be derived from adding up the 13 component scores. The maximum total HEI score is 100 and signifies the highest possible compliance to the DGA-2015. HEI scores were calculated using an average of each participant’s two dietary recalls. Since multiple dietary recalls were used for each participant, scores were calculated by summing across all days per participant before applying the HEI scoring standards and performing further analyses. The simple HEI scoring algorithm method [7], was used as the statistical methodology and scores were calculated using a freely available SAS code [28] developed by the University of Minnesota Nutrition Coordinating Center.

### 2.7. Assessment of Child Food Security

Child food security experiences were measured using a 5-item adapted version of the Child Food Security Assessment (CFSA), which was previously validated for use with children as young as six years [12,15]. One emotional subdomain item “I worry about how hard it is for parents to get enough food” included in the CFSA was removed and replaced with a child food management subdomain item “I tried not to eat a lot so that our food would last” to encompass a broader range of subdomains of child food insecurity. This item tested well in previous validation assessments [15]. All items had high sensitivity and specificity for the domain to which they corresponded to [15]. The items on the adapted CFSA represented four of six previously conceptualized subdomains of child food insecurity (Q1, emotional awareness; Q2–Q3, physical awareness; Q4, initiation of child food management strategies; Q5, cognitive awareness) [29]. A reference frame of “in the last year” was used. Response categories were “a lot, sometimes, or never”. Responses to the CFSA were recoded as follows: “never” = 0, “sometimes” = 1, or “a lot” = 2. Scores were summed (range 0–10) with higher scores indicative of reporting decreased food security. Scores were distributed asymmetrically with a right skew. Four ordinal groups were created that corresponded with summed scores: 0 (high food security), 1 (marginal food security), 2 to 3 (low food security), and 4 to 10 (very low food security) [15]. For analysis, these groups were collapsed to two so that summed scores of 0–1 were representative of food security and 2–10 of food insecurity. 

### 2.8. Covariates

Covariates included in the analysis were sex, age, ethnicity/race (non-Hispanic White, Hispanic, non-Hispanic Black), Supplemental Nutrition Assistance Program (SNAP) participation (provided by the parent), average energy intake, and BMI percentile. 

### 2.9. Statistical Analysis

Descriptive statistics (i.e., mean, standard deviation, number, percentage of sample) were used to describe the sample. Chi square (χ^2^) tests and univariate linear regression models were used to determine if significant differences existed between demographic variables of food secure and insecure children. Mixed effects linear regression models were used to estimate associations between food secure and insecure groups and HEI-2015 total score, with random effects at the school level to account for clustering by schools. Interactions between food insecurity and child ethnicity/race and sex were tested. Separate mixed effects linear regression models were then used to examine associations between food security status and HEI-2015 component scores. All models were adjusted for age, sex, ethnicity/race, SNAP participation, BMI percentile, and energy intake and used robust standard errors to account for heteroscedasticity. All data were analyzed using SPSS Statistics for Macintosh, version 25.0 [30]. 

## 3. Results

### 3.1. Study Sample

Of the 4239 eligible students at the 16 elementary schools, 3303 children (78%) consented to participate in the TX Sprouts study. Out of those consented children, 3137 (95%) completed baseline clinical measures and were included in the clinical trial. The analytic sample included only baseline data from the trial. A random subsample of 738 students completed two 24-h dietary recalls. After removing 26 cases with incomplete survey data for determining food security status and 73 cases with missing ethnicity/race, the sample was 639 students. Furthermore, prior to analysis, 41 cases were removed, as these participants indicated that they were of an ethnicity/race (4 Native American or American Indian; 10 Asian or Pacific Islander; and 27 Mixed or Other Ethnicity) that was too small of a percentage of the total sample to draw conclusions during analysis. These cases were not combined to form a general “other ethnicity” group because they significantly differed in demographic variables and overall diet quality. Previous research has provided evidence of an age-specific relationship between food security and dietary outcomes [4]. It has been recommended to separate samples into age subgroups (1–5 years, 6–11 years, and 12–19 years). While the current study’s population age ranges from 7–13, the sample was not separated as only two participants were above the age of 11 years. The final analytic sample with complete data was 598 students. 

The sample was primarily Hispanic (64%), 55% female, and had an average age of 9.2 ± 0.9 years (range 7–13 years) (Table 1). Food insecurity was reported by 65% of the children. A greater number of food insecure were younger and male. A greater number of Hispanic children were food insecure compared to secure; whereas a greater number of food secure children were more likely to be non-Hispanic White or non-Hispanic Black. A significantly greater number of food insecure children reported receiving SNAP benefits compared to food secure children. Average BMI percentile for the sample was 72.5, and 49.7% of the sample were overweight or obese [21]. There was no significant difference in BMI percentile between food secure and insecure children. There was also no significant difference in energy intake. 

### 3.2. Associations between Food Insecurity and Overall Dietary Quality

After adjustment for sociodemographic characteristics (sex, age, ethnicity/race, and SNAP participation), BMI percentile, and energy intake, food insecurity was associated with lower diet quality scores (β = −3.17; 95% CI = −5.28, −1.06; *p* = 0.003) (Table 2). In this full model, there was a significant association between ethnicity/race and HEI-2015 total score (*p* < 0.001). Compared to non-Hispanic Whites, Hispanic children had 4% higher HEI-2015 totals scores (*p* = 0.004). There was no significant difference between non-Hispanic Whites and Blacks. Higher energy intake was also associated with lower HEI-2015 total scores (*p* = 0.011). There was no significant interaction between food security and sex or food security and ethnicity/race; therefore, the sample was not stratified by sex or ethnicity/race for analysis. Average total HEI-2015 scores between food secure and food insecure children are shown in Table 3. Food secure children vs. food insecure children had higher HEI-2015 total scores (54.5 vs. 52.5; *p* = 0.012). 

### 3.3. Associations between Food Insecurity and Healthy Eating Index-2015 Components

**Figure 1:**
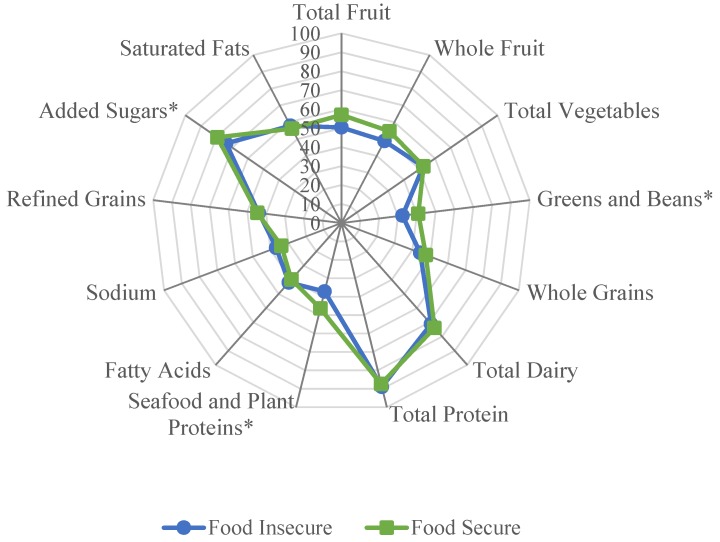
Radar plot visualization of average Healthy Eating Index-2015 component scores contributing to the total score in food insecure and food secure children. Significant differences (*p* < 0.05) in mean component scores between food insecure and food secure children are denoted with an asterisk.

Mixed effects linear regression models were used to compare HEI-2015 component scores between food secure and food insecure children, while adjusting for sociodemographic characteristics, BMI percentile, and energy intake (Table 3). Compared to food secure children, food insecure children had lower component scores for greens and beans (2.32 vs. 1.86, *p* = 0.016), lower mean seafood and plant protein (2.04 vs. 1.62, *p* = 0.006), and lower added sugar (7.95 vs. 7.39, *p* = 0.002). Of note, added sugar is a moderation HEI-2015 component (reverse scoring is applied during calculation of the component score), meaning that a higher component score is representative of lower mean consumption. Figure 1 depicts a radar plot that was constructed to provide a visual representation of the differences of how food secure and food insecure children obtained their overall HEI-2015 scores. Component scores were graphed as percentages (e.g., a total fruit score of 4 out of 5 was graphed as 80%). A perfect HEI-2015 score (100% for each component) would be displayed as a line around the exterior border of the radar plot. 

## 4. Discussion

Diet quality, as measured by the HEI-2015, was lower in food insecure children compared to food secure children. This is the first study to show a relationship between dietary quality and food insecurity while using a child’s own report of their food security experiences. Overall, diet quality for all children was low, but similar to those found in a nationally representative sample of U.S. children (6–11 years) [31]. Previous research has reported that dietary quality differs by ethnicity/race in nationally representative child samples [31,32,33]. This study found that diet quality did not differ by sex but did significantly differ by race/ethnicity. Contrary to our hypothesis; however, the interaction between food insecurity and ethnicity/race was not significant. 

Previous research in studies of children and adolescents have found no association between food insecurity and dietary quality [12,13,14]. Differences from previous studies of children may be attributed to using child report or controlling for a different set of confounding variables that may affect this relationship [34]. Other possible explanations for the novel differences found in this study include whether or not participants utilized food assistance programs. Participation rates in the SNAP by food insecure and food secure children differed within our sample; however, research has found that children’s diets were similar among SNAP participants and low-income nonparticipants. Furthermore, this study only asked about current SNAP participation, reductions or loss of benefits over time may impact dietary intake and may be associated with greater odds of food insecurity [35]. While not measured in this study, food-insecure households are much more likely to use a food pantry or food bank than food-secure households and the nutrient quality of foods obtained at local food banks or food pantries can vary [36,37]. The results of this study were consistent with those in nationally representative samples of adults, which have found that food insecurity is associated with lower dietary quality [38,39]. 

While similar recent studies [13,14] have focused on differences in total HEI score in food secure and insecure children, this study also sought to examine component scores of the HEI-2015 to maximize understanding of dietary quality of patterns of food intake. HEI components can be considered as a set of scores, each measuring alignment with a different aspect of the DGAs and serve as targets for improvements in nutrient density within the diet. Food secure compared to food insecure children had greater intakes of greens and beans, and seafood and plant proteins, and lower intake of added sugar. 

The associations between the greens and beans component and the seafood and plant proteins component and food insecurity found in this study has not been previously reported by others. This may be in part due to the fact that other studies have utilized previous versions of the HEI and as a result may not be directly comparable [7]. For example, in HEI-2015, legumes were allocated to both protein and vegetable components [27]. Therefore, HEI-2015 component scores for total vegetables, greens and beans, and seafood and plant proteins may have been higher than component scores for HEI-2010. Furthermore, much of the existing literature has reported on vegetable intake in general and have found no association with food security status and specific types of vegetable intake [3]. Fram et al. 2015, however, reported that food insecurity was associated with a lower HEI-2005 total vegetable component score in a sample of 9 to 11 year old children [12]. Further research is needed to clarify the relationship between food insecurity and vegetable intake, including type of vegetables, as well as the potential barriers that food insecure households face in access, availability, and utilization of vegetables. 

This study also found a significant association between the added sugar component and food insecurity. Food insecure children had higher intakes of added sugar compared to food secure children. Most previous studies have reported on added sugar intake separately from the HEI. Sharkey et al. found that food insecurity was associated with higher added sugar intake in a convenience sample of Texas children (6–11 years of age) [40]. Fram et al. found that food insecure children compared to food secure children consumed eight grams more of added sugar per day [12]. However, research has also found no association [13] and even an inverse association [41] between food insecurity and added sugar intake in children. Additional research is needed to determine if added sugar intake differs between food insecure and secure children. 

There is evidence suggesting that dietary habits and patterns established during childhood may persist into adulthood [42]. Experiencing food insecurity during critical points in a child’s development may put them at increased risk of chronic diseases. Diets of children within this study, particularly those who were food insecure, strayed from current national dietary recommendations [25]. Early modification of these dietary behaviors in children who are food insecure may promote health and decrease risk of developing chronic diseases over a lifetime [43,44]. Interventions that alleviate the burdens of food insecurity and target improvements in diet quality are needed. 

### Limitations and Strengths

This was a cross-sectional analysis; therefore, no causal relationships could be inferred. However, this analysis used baseline data from an intervention trial, so changes in food security in relation to changes in diet quality can be examined at a later date. Another limitation is that food insecurity is episodic in nature and may be perceived by children differently throughout the year or even month, leading to potential misclassification of food security status. Due to the small sample size and distribution of individuals within food security groupings, this study collapsed the traditional four categories of food security (high, marginal, low, very low) down to food secure (encompassing high and marginal) and food insecure (encompassing low and very low). Self-reported dietary intake is subject to measurement error, bias, and social desirability [45,46,47,48]. In addition, dietary assessment in children poses unique challenges including a potentially limited concept of time, food recognition and knowledge of preparation methods, ability to quantify estimated portion sizes, motivation, literacy, memory capabilities, and concentration span [49,50,51]. However, when measurement error is taken into consideration during interpretation of data, self-report data remain useful and valuable [52]. 

The use of self-report of child food insecurity experiences is seen as a strength of this study. Child-report of their personal experiences with food insecurity has been shown in the literature to be more representative of their actual experiences and rules out potential biases that may result from parental reporting [15,16,17,18,19]. Sample size for this study was smaller than previous studies and is only locally representative; however, the scale of this study enabled us to control for confounding variables of potential relevance to food insecurity and diet, such as body weight or BMI, which prior studies have not controlled for. 

## 5. Conclusions

Poor dietary quality was observed in a low-income, multiethnic sample of 7 to 13 year old children. Significant differences in HEI component scores were observed between food secure and insecure children. Food insecure children had lower overall diet quality and had lower scores for greens and beans, seafood and plant proteins, and added sugar HEI-2015 components. This study contributes to our understanding that dietary intake of food insecure children differs from low-income, food secure children; however, further research is needed to explain why these differences exist. Additional research is needed in large, nationally representative samples where food security status is self-reported by children to better understand the complex interplay between food insecurity and dietary intake. Interventions targeting low-income and food insecure children should investigate methods to improve dietary quality.

## Figures and Tables

**Table 1:** Characteristics of Children by Child-Reported Food-Security Status ^a^.

Characteristics	Total Sample (n = 598)	Food Secure (n = 211)	Food Insecure (n = 387)	*p* Value
	<---- mean ± standard deviation ---- >			
**Age (y)**	9.2 ± 0.9	9.4 ± 0.9	9.1 ± 0.9	<0.001
**BMI Percentile**	72.5 ± 28.2	72.4 ± 29.7	72.6 ± 28.6	0.941
**Energy (kcal)**	1465 ± 539	1450 ± 483	1473 ± 567	0.621
	<---- n (%) ---->			
**Sex**				0.012
Male	268 (44.8)	80 (37.9)	188 (48.6)	
Female	330 (55.2)	131 (62.1)	199 (51.4)	
**Ethnicity/Race**				0.001
Non-Hispanic White	139 (23.2)	66 (31.3)	73 (18.9)	
Hispanic	381 (63.7)	115 (54.5)	266 (68.7)	
Non-Hispanic Black	78 (13.0)	30 (14.2)	48 (12.4)	
**SNAP Participation**				0.014
Yes	179 (29.9)	50 (23.7)	129 (33.3)	
No	419 (70.1)	161 (76.3)	258 (66.7)	

Abbreviations: kcal, kilocalories; BMI, body mass index; SNAP, Supplemental Nutrition Assistance Program. ^a^ *p*-values were from χ^2^ tests and univariate linear regression models.

**Table 2:** Mixed effects linear regression model of food security status and Healthy Eating Index-2015.

	Unstandardized β	Standard Error	95% Confidence Interval for β	*p*-Value
**Age**	−0.05	0.05	−0.15, 0.06	0.362
**Sex**				0.200
Male	Referent	---	---	----
Female	−2.52	1.69	−5.84, 0.78	0.134
**Ethnicity/Race**				<0.001
Non-Hispanic White	Referent	---	---	----
Hispanic	3.79	1.29	1.22, 6.30	0.004
Non-Hispanic Black	−0.347	2.89	−4.89, 4.14	0.879
**Energy (kcal)**	−0.003	0.001	−0.006, −0.001	0.011
**SNAP Participation (yes)**				0.200
Yes	Referent	---	---	----
No	1.6	1.25	−0.85, 4.05	0.200
**BMI Percentile**	−0.03	0.001	−0.06, 0.01	0.105
**Child-Level Food Security**				0.005
Food Secure	Referent	---	---	----
Food Insecure	−3.17	1.08	−5.28, −1.06	0.003
**Food Security x Sex Interaction**				0.194
Male	Referent	---	---	----
Female	2.05	1.60	−1.08, 5.19	0.126
**Food Security x Ethnicity Interaction**				0.287
Non-Hispanic White	Referent	---	---	----
Hispanic	−3.19	2.52	−8.13, 1.75	0.205
Non-Hispanic Black	−4.37	2.78	−9.83, 1.09	0.116

Abbreviations: kcal, kilocalories; SNAP, Supplemental Nutrition Assistance Program; BMI, Body Mass Index.

**Table 3:** Mixed effects linear regression model of food security status and Healthy Eating Index-2015 and component scores ^a^.

				Means		
Parameter	Unstandardized β	Standard Error	95% Confidence Interval for β	Food Secure	Food Insecure	*p*-Value
HEI Total Score	−1.98	0.79	−3.52, −0.43	54.48	52.50	0.012
Total Vegetables	−0.01	0.14	−0.28, 0.26	2.63	2.62	0.940
Greens and Beans	−0.42	0.15	−0.72, −0.12	2.04	1.62	0.006
Total Fruit	−0.33	0.17	−0.67, 0.01	2.85	2.52	0.059
Whole Fruit	−0.29	0.19	−0.67, 0.09	2.73	2.44	0.132
Whole Grains	−0.32	0.27	−0.89, 0.20	4.76	4.44	0.223
Total Dairy	−0.27	0.30	−0.86, 0.32	7.39	7.12	0.363
Total Protein	0.09	0.10	−0.10, 0.27	4.37	4.45	0.378
Seafood and Plant Protein	−0.46	0.19	−0.84, −0.09	2.32	1.86	0.016
Fatty Acids	0.20	0.34	−0.46, 0.87	3.98	4.19	0.546
Sodium	0.29	0.17	−0.05, 0.63	3.39	3.68	0.095
Refined Grains	−0.08	0.32	−0.71, 0.55	4.47	4.39	0.802
Added Sugar	−0.56	0.18	−0.92, −0.21	7.95	7.39	0.002
Saturated Fat	0.17	0.27	−0.35, 0.69	5.62	5.79	0.521

^a^ Food secure was the referent group. Models controlled for sex, age, ethnicity/race (non-Hispanic White, Hispanic, non-Hispanic Black), Supplemental Nutrition Assistance Program (SNAP) participation, average energy intake, and BMI percentile.
